# Response to single agent PD-1 inhibitor after progression on previous PD-1/PD-L1 inhibitors: a case series

**DOI:** 10.1186/s40425-017-0273-y

**Published:** 2017-08-15

**Authors:** Dylan J. Martini, Aly-Khan A. Lalani, Dominick Bossé, John A. Steinharter, Lauren C. Harshman, F. Stephen Hodi, Patrick A. Ott, Toni K. Choueiri

**Affiliations:** 1 Lank Center for Genitourinary Oncology, Dana-Farber Cancer Institute, Harvard Medical School, 450 Brookline Ave D1230, Boston, MA 02215 USA; 2 Melanoma Center, Dana-Farber Cancer Institute, Harvard Medical School, Boston, MA USA; 3 Center for Immuno-Oncology, Dana-Farber Cancer Institute, Harvard Medical School, 450 Brookline Ave, Boston, MA 02215 USA

**Keywords:** PD-1/PD-L1 inhibitor, Sequential treatment, Immune checkpoint blockade, Case reports

## Abstract

**Background:**

Monoclonal antibodies targeting the PD-1/PD-L1 axis have gained increasing attention across many solid tumors and hematologic malignancies due to their efficacy and favorable toxicity profile. With more than 1 agent now FDA-approved in a wide variety of tumor types, and with others in clinical trials, it is becoming more common that patients present to clinic for potential treatment with a second PD-1/PD-L1 inhibitor.

**Case presentation:**

In this report, we present two patients with renal cell carcinoma and one with melanoma who received PD-1/PD-L1 inhibitors. Upon progression on their first-line PD-1/PD-L1 inhibitors, these patients received a different PD-1 inhibitor (nivolumab in all cases) and all had progressive disease as their best response to the subsequent PD-1 inhibitor. The reported clinical information focuses on the course of the disease and the responses to all treatment regimens.

**Conclusions:**

Clinicians should refrain from using multiple PD-1/PD-L1 inhibitors sequentially outside of clinical trials until there is sufficient data to support this practice routinely. Prospective studies that allow prior treatment with PD-1/PD-L1 are needed to validate the efficacy and safety of these drugs in the second line or later setting. Furthermore, ongoing efforts that aim to identify mechanisms of resistance to immunotherapy will be informative and may ultimately assist physicians in select the optimal treatment following progression on PD-1/PD-L1 inhibitor.

## Background

The programmed cell death protein-1 (PD-1) and its ligands, PD-L1 and PD-L2, are part of a pathway that cancer cells utilize to evade immune surveillance [1]. Monoclonal antibodies targeting the PD-1/PD-L1 axis have demonstrated efficacy in numerous malignancies [2–16]. Five of these agents (atezolizumab, nivolumab, pembrolizumab, avelumab, and durvalumab) have gained United States Food and Drug Administration (FDA) approval for the treatment of non-small cell lung cancer (NSCLC), renal cell carcinoma (RCC), melanoma, urothelial carcinoma, head and neck squamous cell carcinoma (HNSCC), Hodgkin’s Lymphoma, and Merkel cell carcinoma [17]. With these agents and others in development, physicians are more commonly faced with the question of whether to treat patients with sequential PD-1 blockade. While there is a general acceptance that these drugs are “similar”, there are some subtle differences among them and many clinicians wonder if a subsequent different PD-1 inhibitor can of be of any help to patients with few therapeutic options after progression. The outcomes of these patients in this expanding clinical setting are largely unknown and have not been assessed in clinical trials. In this report, we present the cases of three patients (two with metastatic RCC and one with melanoma) who initially responded to PD-1/PD-L1 blockade before progressing and later immediately progressed upon re-treatment with a different PD-1 inhibitor.

## Case presentations

### Case presentation 1

A 54-year-old man underwent a radical nephrectomy which revealed an 11.5 cm pT2bN0M0 clear cell renal cell carcinoma (ccRCC) on pathologic review. Four years later, he was diagnosed with metastatic disease to the lungs and hilar lymph nodes and began treatment with an anti-PD-L1-based combination. The patient had a best response of stable disease with tumor shrinkage and remained on treatment for 15 months. He discontinued therapy for progressive disease to the sacrum and the cerebellum and subsequently underwent stereotactic radiosurgery to the brain. Approximately 7 weeks after the last dose of the anti-PD-L1-based combination, he initiated treatment with 3 mg/kg nivolumab monotherapy every 2 weeks. After the patient received 4 doses, imaging showed progressive disease in the lung and hilar lymph node after 7 weeks.

### Case presentation 2

A 67-year-old male was diagnosed with pT2aN0M1 ccRCC with multiple subcentimeter metastases to the lungs. The patient initially underwent metastatectomy to remove a 0.6 cm tumor in the left upper lobe, but he experienced progression 1 year later. An anti-PD-L1-based combination was started and he had stable disease as best response with tumor shrinkage and remained on treatment for 8 months until he discontinued therapy for new liver metastases. He then progressed after 2 cycles of axitinib. The patient received 8 doses of 3 mg/kg nivolumab monotherapy every 2 weeks, 6 months after the last dose of the anti-PD-L1-based combination. He experienced disease progression in the lung, lymph nodes, and liver after 4 months.

### Case presentation 3

A 78-year-old gentleman was diagnosed with stage IVM1c BRAF^V600^mutant cutaneous melanoma with metastases to the kidney, adrenal, and lymph node. The patient began treatment with a vemurafenib, a BRAF inhibitor, but discontinued after two months for progressive disease. He then progressed through treatment with cytotoxic chemotherapy, ipilimumab, and a combination of anti-BRAF/MEK (dabrafenib plus trametinib) combination therapy. He later received therapy with anti-PD-1 pembrolizumab for 5 months before being discontinued for treatment-related insulin-dependent diabetes mellitus, chronic pruritus, and joint pain. The patient had a complete response on treatment. Approximately 20 months after the patient’s last dose of pembrolizumab, he progressed and initiated nivolumab every 2 weeks for 3 doses. Although the patient continued on insulin for treatment of diabetes mellitus throughout his time on nivolumab, neither the pruritus nor the joint pain recurred after treatment with nivolumab. Treatment was discontinued after 7 weeks due to disease progression in the bilateral adrenal lesions, lymph nodes, and a new lesion in the liver.

## Discussion

Given the approval of different PD-1 and PD-L1 inhibitors in several solid tumor and hematological malignancies as of May 2017, it is anticipated that clinical decisions facing patients and physicians as described above will become more common. For example, there are already two PD-1 inhibitors, pembrolizumab and nivolumab, approved for NSCLC and melanoma in both the first and second-line setting. The shifting treatment paradigms in NSCLC and other diseases such as melanoma and RCC are complicated by the paucity of data for patients who fail PD-1/PD-L1 inhibitors. This is partly because randomized trials in which these agents were investigated in the second-line setting or beyond, did not allow inclusion of patients who had prior treatment with a PD-1 or PD-L1 inhibitor [3–10, 12, 13, 15, 18]. Currently, there are numerous ongoing and pending trials in RCC, melanoma, and NSCLC that allow prior treatment with a PD-1/PD-L1 inhibitor (Table 1). As many of these agents target the same PD-1/PD-L1 axis, development of resistance to one agent may plausibly lead to class resistance and, therefore, the likelihood of response to a second monotherapy agent given from the same class could be low.

**Table 1:** Select ongoing and pending clinical trials that allow prior treatment with a PD-1 or PD-L1 inhibitor

Clinicaltrials.gov identifier	Experimental agents	Regimen targets	Phase	Tumor type
NCT02899078	Nivolumab + Ibrutinib	PD-1 + BTK	1b/2	RCC
NCT02923531	Nivolumab + X4P-001	PD-1 + CXCR4	1b/2a	RCC
NCT02963610*	Pembrolizumab + Lenalidomide	PD-1 + immunomodulatory	1/2	NSCLC
NCT03083808	Pembrolizumab + Docetaxel/Pemetrexed/Gemcitabine	PD-1 + Chemotherapy	2	NSCLC
NCT03041181	Nivolumab + Docetaxel	PD-1 + Chemotherapy	2	NSCLC
NCT02437136	Pembrolizumab + Entinostat	PD-1 + HDAC	1b/2	NSCLC and melanoma
NCT02959437	Pembrolizumab + Azacitidine + Epacadostat	PD-1 + Chemotherapy + IDO-1	1/2	NSCLC**
NCT03084640	Pembrolizumab + CMP-001	PD-1 + TLR9	1b/2	melanoma
NCT03014648	Atezolizumab	PD-L1	2	NSCLC

PD-1 programmed cell death protein 1, BTK: Bruton’s tyrosine kinase, RCC: renal cell carcinoma, CXCR4: C-X-C chemokine receptor type 4, NSCLC: non-small cell lung cancer, HDAC: histone deacetylases, IDO-1: Indoleamine 2,3-Dioxygenase 1, TLR9: Toll-like receptor 9, PD-L1 programmed death-ligand 1

*Prior treatment with anti-PD-1/PD-L1 is only allowed for the Phase 2 portion of this trial (which is only for NSCLC patients)

**Only the NSCLC cohort allows prior treatment with PD-1/PD-L1

Please note that this table was constructed using the following search terms on clinicaltrials.gov on 5/12/2017: “Nivolumab AND previously treated”, “Pembrolizumab AND previously treated”, and “Atezolizumab AND previously treated”. After generating a list of trials, the eligibility criteria for each trial was manually screened for inclusion in this table

When successful, PD-1 blockade establishes a favorable equilibrium between the immune response to, and immune evasion by, cancer cells [19]. In some situations, patients who achieve an objective response on treatment eventually experience disease progression. This acquired resistance to immunotherapy, also known as secondary tumor immune escape, has many plausible immunological explanations such as decreased intratumoral T-cell infiltration, suboptimal T-cell activation, upregulation of additional immune-checkpoints, or loss of tumor immunogenicity [20, 21]. A recent genomic analysis comparing pretreatment and progression biopsy samples from melanoma patients who initially experienced an objective response on anti-PD-1 therapy showed that resistance was associated with: diminished antigen-presentation due to a defective beta-2-microglubulin (B2M) protein or impaired interferon-receptor signaling due to truncated Janus Kinase 1 (JAK1) or Janus Kinase 2 (JAK2) proteins [22]. Another study comparing differentially expressed genes between responders and non-responders to immunotherapy in melanoma showed that non-responders express a transcriptional signature exhibiting simultaneous upregulation of genes involved in the regulation of angiogenesis, cell adhesion, mesenchymal transition, extracellular matrix (ECM remodeling), and wound-healing [23].

Although many patients experience resistance after responding to PD-1 or PD-L1 inhibitors, there may be situations where re-treatment has plausible rationale. Re-treatment with PD-1 blockade may be especially effective in patients who discontinue the first PD-1 or PD-L1 inhibitor for reasons other than disease progression. In fact, successful re-introduction of anti-PD-1 therapy after delayed tumor recurrence has been reported in one patient with melanoma [24]. Data have shown that even similar PD-1 agents, such as pembrolizumab and nivolumab, target different epitopes of PD-1 [25]. It is also reasonable to treat a patient with a PD-1 inhibitor after a PD-L1 inhibitor, or vice versa, given that the PD-1 pathway has 3 inhibitory interactions (PD-1:PD-L1, PD-1:PD-L2, and PD-L1:B7.1) and each of these drugs only inhibit two of these [20]. A melanoma mouse model has shown that there is a synergistic effect of combining PD-1 and PD-L1 inhibition [26]. There is also an ongoing clinical trial testing the combination of PD-1 and PD-L1 inhibition [NCT02118337].

There are numerous opportunities to address mechanisms of resistance to immunotherapy other than retreatment with PD-1/PD-L1 monotherapy. For example, anti-Vascular Endothelial Growth Factor (VEGF) therapy has been shown to increase T-cell infiltration [27] and preclinical data suggests that antibodies blocking T-cell co-stimulatory proteins [28–30] or low-dose Interleukin-2 (IL-2) [31] may augment the immune response to anti-PD-1/PD-L1. Currently, clinical trials are ongoing which investigate Lymphocyte-activation gene 3 (LAG-3) and T-cell immunoglobulin and mucin-domain containing-3 (TIM-3) blockade either as monotherapy or in combination with an anti-PD-1 agent [NCT01968109, NCT02676869, NCT02817633, NCT02936102, NCT02966548, NCT02460224, NCT03005782]. There is also an early clinical trial investigating the synergy of IL-2 and PD-1 inhibition in metastatic RCC [NCT02989714]. Additionally, stereotactic radiation therapy has been shown to increase the repertoire of antigen-specific T-cells and antitumor antibodies, which may increase the likelihood of the “abscopal effect” [32]. This suggests a possible synergy between radiotherapy and immunotherapy, which is being investigated in several ongoing clinical trials [NCT02821182, NCT02608385, NCT03050554, NCT02407171, NCT02843165, NCT02837263].

However, it is unlikely possible to provide clinical trials for all potential immunotherapy sequences. Therefore, databases that captures real-life clinical data on the safety and efficacy of anti-PD-1/PD-L1 therapy after treatment with another PD-1/PD-L1 inhibitor may potentially help to better characterize the outcomes of these patients in an unselected setting. For example, there is a United States-based registry for patients treated with high-dose IL-2, Proleukin® Observational Registry to Evaluate the Treatment Patterns and Clinical Response in Malignancy (PROCLAIM), which adds real-world experience for patients treated with IL-2 [33]. Similar registries for anti-PD-1/PD-L1 would also be beneficial in the current immunotherapy era.

## Conclusions

Currently, there is insufficient data to support treatment with a different PD-1/PD-L1 inhibitor after progression on PD-1 pathway blockade, and such practice should be discouraged. Ideally, prospective studies that investigate the efficacy and safety of a PD-1/PD-L1 inhibitor in the second-line setting or later, which allows prior treatment with one of these agents, are needed to validate the use of sequential PD-1 blockade. Combined strategy such as dual immune agents or combination of PD-1/PD-L1 non-immune therapy appears to be more supported by preclinical data and such trials represent a better opportunity to improve outcomes. Furthermore, ongoing efforts that aim to identify mechanisms of resistance to immunotherapy will be informative and may ultimately assist physicians in selecting a rational approach to the initial, and sequential, treatment options for these patients.

## Abbreviations

B2M: Beta-2-microglubulin protein
ccRCC: Clear cell renal cell carcinoma
ECM: Extracellular matrix
FDA: United States Food and Drug Administration
HNSCC: Head and neck squamous cell carcinoma
IL-2: Interleukin-2
LAG-3: Lymphocyte-activation gene 3
NSCLC: Non-small cell lung cancer
PD-1: Programmed cell death protein-1
PD-L1: Programmed death ligand-1
PD-L2: Programmed death ligand-2
PROCLAIM: Proleukin® Observational Registry to Evaluate the Treatment Patterns and Clinical Response in Malignancy
RCC: Renal cell carcinoma
TIM-3: T-cell immunoglobulin and mucin-domain containing-3
VEGF: Vascular endothelial growth factor
